# Acute effects of intravenous DMT in a randomized placebo-controlled study in healthy participants

**DOI:** 10.1038/s41398-023-02477-4

**Published:** 2023-05-23

**Authors:** Severin B. Vogt, Laura Ley, Livio Erne, Isabelle Straumann, Anna M. Becker, Aaron Klaiber, Friederike Holze, Anja Vandersmissen, Lorenz Mueller, Urs Duthaler, Deborah Rudin, Dino Luethi, Nimmy Varghese, Anne Eckert, Matthias E. Liechti

**Affiliations:** 1 Clinical Pharmacology and Toxicology, Department of Biomedicine and Department of Clinical Research, University Hospital Basel, Basel, Switzerland; 2 Department of Pharmaceutical Sciences, University of Basel, Basel, Switzerland; 3 Psychiatric University Hospital, University of Basel, Basel, Switzerland; 4 Transfaculty Research Platform Molecular and Cognitive Neuroscience, University of Basel, Basel, Switzerland

**Keywords:** Clinical pharmacology, Neuroscience, Psychology

## Abstract

*N,N*-dimethyltryptamine (DMT) is distinct among classic serotonergic psychedelics because of its short-lasting effects when administered intravenously. Despite growing interest in the experimental and therapeutic use of intravenous DMT, data are lacking on its clinical pharmacology. We conducted a double-blind, randomized, placebo-controlled crossover trial in 27 healthy participants to test different intravenous DMT administration regimens: placebo, low infusion (0.6 mg/min), high infusion (1 mg/min), low bolus + low infusion (15 mg + 0.6 mg/min), and high bolus + high infusion (25 mg + 1 mg/min). Study sessions lasted for 5 h and were separated by at least 1 week. Participant’s lifetime use of psychedelics was ≤20 times. Outcome measures included subjective, autonomic, and adverse effects, pharmacokinetics of DMT, and plasma levels of brain-derived neurotropic factor (BDNF) and oxytocin. Low (15 mg) and high (25 mg) DMT bolus doses rapidly induced very intense psychedelic effects that peaked within 2 min. DMT infusions (0.6 or 1 mg/min) without a bolus induced slowly increasing and dose-dependent psychedelic effects that reached plateaus after 30 min. Both bolus doses produced more negative subjective effects and anxiety than infusions. After stopping the infusion, all drug effects rapidly decreased and completely subsided within 15 min, consistent with a short early plasma elimination half-life (t_1/2α_) of 5.0–5.8 min, followed by longer late elimination (t_1/2β_ = 14–16 min) after 15–20 min. Subjective effects of DMT were stable from 30 to 90 min, despite further increasing plasma concentrations, thus indicating acute tolerance to continuous DMT administration. Intravenous DMT, particularly when administered as an infusion, is a promising tool for the controlled induction of a psychedelic state that can be tailored to the specific needs of patients and therapeutic sessions.

**Trial registration:** ClinicalTrials.gov identifier: NCT04353024

## Introduction

*N,N*-dimethyltryptamine (DMT) is a naturally occurring psychedelic that is a component of Ayahuasca, which is widely used for recreational and spiritual purposes [1]. Ayahuasca contains plant-derived monoamine oxidase (MAO) inhibitors [2], increasing the bioavailability of orally administered DMT and slowing its metabolism. When administered orally without an MAO inhibitor, DMT is not psychoactive because of its rapid presystemic and systemic metabolism. However, DMT alone is psychoactive when administered parenterally, thereby avoiding enteral and hepatic first-pass metabolism. Several studies have investigated the acute effects of DMT alone when administered intravenously [3–10], intramuscularly [11–14], and by inhalation [2]. These forms of administration allow the use and study of DMT alone without potentially additive psychoactive effects of MAO inhibitors. Importantly, intravenous administration allows the rapid and controlled induction of a psychedelic experience and thus may be a therapeutically particularly interesting form of use of a classic psychedelic. For example, a recent study showed rapid antidepressant effects in patients with treatment-resistant major depressive disorder after the administration of intravenous DMT, with acute effects that lasted only 30 min [10]. The first studies with DMT or similar chemical analogs began in the 1950s, which used intramuscular administration to mimic psychotic states in healthy volunteers and patients [12, 13, 15–17]. Intravenous DMT administration was subsequently investigated in the 1990s [6, 8, 9]. DMT was administered at doses up to 0.4 mg/kg as an intravenous bolus over 45 s. DMT was found to be fully hallucinogenic at doses of 0.2 and 0.4 mg/kg (~15 and 30 mg, respectively) [8, 9]. A later study administered doses of 0.2 or 0.3 mg/kg (~15 or 25 mg) over 5 min, followed by a break of 1 min and a continuous infusion with 0.015 mg/kg (~1 mg)/min or 0.02 mg/kg (~1.5 mg)/min, respectively, over 84 min [3]. Two recent studies investigated DMT administration as a single intravenous bolus. Intravenous bolus DMT administration (7–20 mg or 0.1 and 0.3 mg/kg) produced rapid and short-lasting but also overwhelming subjective effects [5, 10, 18, 19], referred to by some subjects as near-death experiences [5]. A regimen with a loading dose as a bolus and a infusion administration model has also been theoretically developed [4], with the goal of inducing a prolonged and immersive DMT psychedelic experience. The authors proposed to combine a 25 mg (0.3 mg/kg) DMT bolus over 30 s with an infusion [4], but the model has not yet been tested in humans. Despite interest in the experimental and therapeutic use of DMT, data are lacking on the clinical pharmacology of DMT, including acute subjective effects, tolerability, and pharmacokinetics of different doses and applications of intravenous DMT. Therefore, the present study sought to characterize different administration regimens of intravenous DMT that were designed to induce a psychedelic state over 90 min. The dosing regimens included two different continuous infusion doses (0.6 and 1 mg/min over 90 min) and combinations of initial loading bolus doses (15 or 25 mg), followed by 90-min DMT infusions as proposed previously [4]. We repeatedly assessed subjective and cardiovascular effects during the subjects’ experience over time and thereafter. We also repeatedly sampled blood and determined plasma levels of DMT [20] to characterize the pharmacokinetics of intravenous DMT.

DMT induces its subjective effects primarily via the activation of serotonin 5-hydroxytryptamine 2A receptors, similar to other classic psychedelics, including lysergic acid diethylamide (LSD) and psilocybin [21]. LSD and psilocybin-induced endocrine effects, including increases in circulating oxytocin [22–24] and brain-derived neurotrophic factor (BDNF) [23, 25, 26], that may contribute to their therapeutic effects or be useful as biomarkers in patients [27]. Moreover, in vitro and animal studies demonstrated the neuroplasticity-promoting properties of psychedelics, including DMT [28]. Therefore, we also determined whether DMT administration acutely alters plasma levels of oxytocin and BDNF.

## Methods and Materials

### Study design

The study used a double-blind, placebo-controlled, crossover design with five experimental test sessions to investigate responses to (*i*) placebo, (*ii*) low infusion (0.6 mg/min), (*iii*) high infusion (1 mg/min), (*iv*) low infusion (0.6 mg/min) + low bolus (15 mg), and (*v*) high infusion (1 mg/min) + high bolus (25 mg) of DMT. The order of administration was random and counterbalanced. The washout periods between sessions were at least one week. The study was conducted in accordance with the Declaration of Helsinki and International Conference on Harmonization Guidelines in Good Clinical Practice and were approved by the Ethics Committee of Northwest Switzerland and Swiss Federal Office for Public Health. The study was registered at ClinicalTrials.gov (NCT04353024).

### Participants

Twenty-seven healthy participants (12 men and 15 women; mean age 33 years; range: 25–58 years) were recruited by word of mouth or from a pool of volunteers who had contacted our research group because they were interested in participating in a clinical trial that investigated psychedelics. All of the subjects provided written informed consent and were paid for their participation. Exclusion criteria were age < 25 years or > 65 years, pregnancy (urine pregnancy test at screening and before each test session), personal or family (first-degree relative) history of major psychiatric disorders (assessed by the Semi-structured Clinical Interview for *Diagnostic and Statistical Manual of Mental Disorders*, 4th edition, Axis I disorders by a trained psychologist or physician), the use of medications that may interfere with the study medications (e.g., antidepressants, antipsychotics, and sedatives), chronic or acute physical illness (e.g., abnormal physical exam, electrocardiogram, or hematological and chemical blood analyses), tobacco smoking (>10 cigarettes/day), the lifetime prevalence of hallucinogenic substance use > 20 times, illicit drug use within the last 2 months, and illicit drug use during the study period (determined by urine drug tests). The participants were asked to consume no more than 10 standard alcoholic drinks/week and have no more than one drink on the day before the test sessions. Twenty-four participants had previously used a psychedelic, including LSD (15 participants, 1–6 times), psilocybin (15 participants, 1–6 times), and DMT (3 participants, 1–6 times). Twenty-one participants had used methylenedioxymethamphetamine (1–20 times). Nineteen participants had previously used a stimulant, including methylphenidate (three participants, once), amphetamine (eight participants, 1–15 times), and cocaine (10 participants, 1–2 times). Three participants had used 4-bromo-2,5-dimethoxyphenethylamine (1–2 times). Three participants had used ketamine (1–4 times).

### Study drug

DMT hemifumarate (99.9% high-performance liquid chromatography purity, ReseaChem GmbH, Burgdorf, Switzerland) was formulated according to good manufacturing practice in sterile units that contained 5 mg/ml (vials for the bolus dose) and 18 mg/ml (vials for the infusion) of DMT in 1 ml of purified water. 5 mg of DMT hemifumarate contains 3.82 mg of DMT freebase and is equivalent to 6.2 mg of DMT fumarate. The exact analytically confirmed DMT hemifumarate content (mean ± SD) was 4.83 ± 0.01 mg (*n* = 10 samples) for the 5 mg vials and 17.91 ± 0.12 mg (*n* = 10 samples) for the 18 mg vials. The respective DMT free base content was 3.69 ± 0.1 and 13.69 ± 0.09 mg, respectively. The stability of DMT products was again confirmed after the study ended. Placebo consisted of identical sterile units that were filled with purified water only. For each session, the content of five vials that contained either 5 mg/ml DMT or placebo was diluted with saline solution (0.9% NaCl) to a volume of 20 ml. In the same way, the content of five 18 mg/ml DMT or placebo vials was diluted with saline solution (0.9% NaCl) to a volume of 50 ml into an infusion pump syringe. Each subject received an intravenous bolus dose of 20 ml that was constantly administered over 45 s at the beginning of the study session (t = 0 min), followed by a continuous infusion of 50 ml over 90 min, starting at t = 1 min and using an infusion pump from Fresenius Kabi (Injectomat Agilia D) with a 50 ml perfusor syringe from Brown. At the end of each session and at the end of the study, the participants were asked to retrospectively guess their treatment assignment to evaluate blinding.

### Study procedures

The study included a screening visit, five 4-h test sessions, and an end-of-study visit. Test days were separated by at least one week. The sessions were conducted in a calm hospital room. Only one research subject and two investigators were present during each test session. The test sessions began at 8:00 AM or 1:00 PM, with starting times that remained consistent within each participant across all five sessions. A urine sample was taken to verify abstinence from drugs of abuse, and a urine pregnancy test was performed in women prior to each session. The subjects then underwent baseline measurements. DMT or placebo was administered at 9:00 AM for the session that began at 8:00 AM or at 2:00 PM for the session that began at 1:00 PM. The outcome measures were repeatedly assessed for 150 min. The subjects were sent home approximately 15 min after the last measurement.

### Subjective drug effects and effect durations

Subjective effects were assessed repeatedly using subjective effect scales 1 h before and 0, 2, 3.5, 5, 7.5, 10, 12.5, 15, 20, 30, 40, 50, 60, 70, 80, 90, 92.5, 95, 100, 105, 110, 120, 130, 140, and 150 min after starting the drug administration. Subjective effect scales included verbal ratings of “any drug effect,” “good drug effect,” “bad drug effect,” and “anxiety” (Likert scale; 0 to 10 for no to maximal effect). The Adjective Mood Rating Scale [29] was used 1 h before and at the end of the session (150 min). The 5 Dimensions of Altered States of Consciousness (5D-ASC) scale [30, 31] was used as the primary outcome measure and was administered 150 min after drug administration to retrospectively rate peak drug effects. Mystical experiences were assessed 150 min after drug administration using the States of Consciousness Questionnaire (SOCQ) [32, 33] that includes the 30-item Mystical Effects Questionnaire (MEQ30) [34] and subscales for “Aesthetic experience” and negative “Nadir effects.” Subjective effect measurements are described in detail in the Supplementary Methods online.

### Autonomic and adverse effects

Blood pressure and heart rate were measured twice each time at baseline and 2, 5, 10, 15, 20, 30, 40, 50, 60, 70, 80, 90, 95, 100, 105, 110, 120, 130, 140, and 150 min after drug administration. Adverse effects were assessed 1 h before and 150 min after drug administration using the List of Complaints [35].

### Endocrine effects and plasma BDNF levels

Plasma concentrations of oxytocin and BDNF were determined as previously described [23, 24]. Oxytocin was measured before and 60 min after drug administration. Plasma BDNF levels were measured at baseline and 90 and 150 min after drug administration.

### Plasma DMT concentrations

Blood was collected into lithium heparin tubes. The blood samples were immediately centrifuged, and plasma was subsequently stored at −80 °C until analysis. Plasma concentrations of DMT and metabolites were determined by high-performance liquid chromatography–tandem mass spectrometry using a validated method as previously described [20].

### Pharmacokinetic analyses

Pharmacokinetic parameters were estimated using non-compartmental methods in Phoenix WinNonlin 8.3 (Certara, Princeton, NJ, USA) as described previously [36].

### Data analysis

Peak (E_max_) or peak change from baseline (ΔE_max_) values were determined for repeated measures. The values were then analyzed using analysis of variance (ANOVA), with the drug as the within-subjects factor, followed by the Tukey *post hoc* test, using R 4.2.1 software. The criterion for significance was *p* < 0.05. Order effects were excluded using ANOVA with session order as factor.

## Results

### Subjective drug effects

**Fig. 1:**
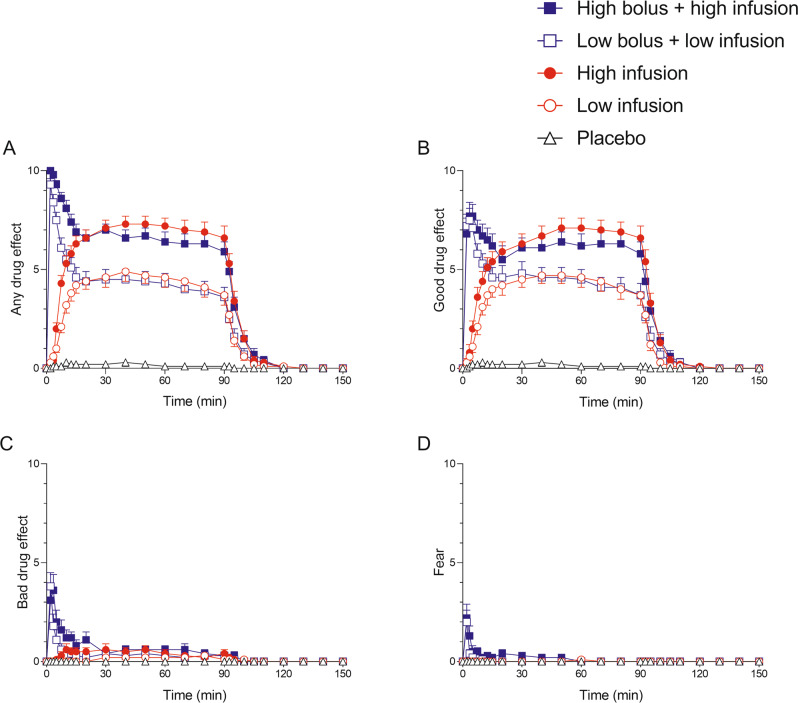
Acute subjective effects of *N,N*-dimethyltryptamine (DMT) over time. The different intravenous dosing conditions of DMT were the following: Low infusion (0.6 mg/min), high infusion (1 mg/min), low bolus (15 mg) + low infusion (0.6 mg/min), and high bolus (25 mg) + high infusion (1 mg/min). The low and high bolus doses induced similar maximal drug effects on all four subjective effect scales, peaking within the first 2 min after intravenous bolus administration and decreasing relatively quickly within 10–15 min. The low and high infusions without a bolus dose induced “any drug effects” (**A**) and “good drug effects (**B**),” reaching a plateau after 30 min and remaining stable at the respective level until the end of the infusion at 90 min. After stopping the infusion, all drug effects rapidly and completely subsided within 15 min. Only minimal “bad drug effects (**C**)” and no “anxiety (**D**)” were induced by the infusions. Effects of the low and high bolus doses did not differ significantly, whereas the different infusion rates produced significantly different plateaus of “any drug effects” and “good drug effects.” Bolus doses were administered at t = 0. Infusions started at t = 1 min and lasted until t = 90 min. The data are expressed as the mean ± SEM in 27 participants. The corresponding maximal responses and statistics are shown in Table 1 and Supplementary Table S5.

**Fig. 2:**
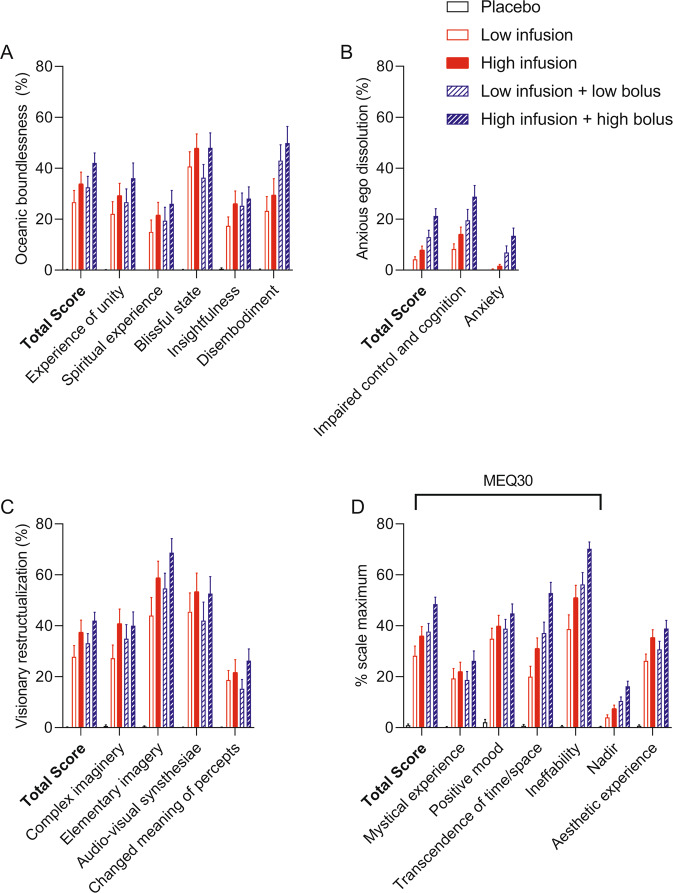
Acute subjective effects of *N,N*-dimethyltryptamine (DMT) rated after the session. Acute alterations of mind on the 5 Dimensions of Altered States of Consciousness (5D-ASC) scale (**A**–**C**) and acute mystical-type experiences on the Mystical Experience Questionnaire 30 (MEQ30) induced by DMT (**D**). The high bolus + high infusion induced the highest scores on all subscales. The data are expressed as the mean ± SEM percentage of maximally possible scale scores in 27 participants. Statistics are shown in Supplementary Table S1.

Subjective effects over time on the subjective effect scales are shown in Fig. 1. Statistics are summarized in Table 1 and Supplementary Table S1, and S5. The low and high bolus doses rapidly induced very intense psychedelic effects and similar maximum effects on all four subjective effect scales (Fig. 1). Subjective effects peaked within the first 2 min after bolus administration. Drug effects that were induced by the high bolus dose lasted 5–10 min longer than the low bolus dose. Both bolus doses were relatively well tolerated, reflected by overall higher positive than negative subjective drug effects and few adverse effects on the List of Complaints (Table 1). Nevertheless, both bolus doses produced very intense and overwhelming responses and more negative subjective effects and anxiety than the infusions (Fig. 1, Table 1, Supplementary Table S5). The DMT infusions without a bolus dose induced slowly increasing psychedelic effects that reached plateaus after 30 min. The higher infusion rate induced greater “any drug effects” (*p* < 0.001) and “good drug effects” (*p* < 0.01) compared with the low infusion rate. The infusion of DMT did not produce negative drug effects or anxiety compared with placebo. After stopping the infusion, all drug effects rapidly decreased and completely subsided within 15 min. Alterations of mind and mystical-type effects are shown in Fig. 2. All DMT conditions induced pronounced psychedelic effects, including mystical experiences, compared with placebo, reflected by high ratings on the 5D-ASC and MEQ (Fig. 2, Supplementary Table S1). The higher dose conditions generally produced greater psychedelic peak effects compared with the lower dose infusion conditions (Fig. 2, Supplementary Table S1). The high bolus dose led to more negative peak effects compared with the other conditions, reflected by significantly higher ratings of “Anxious Ego Dissolution” on the 5D-ASC and “Nadir effects” on the MEQ (Fig. 2, Supplementary Table S1).

**Table 1:** Mean values and statistics for the acute subjective and autonomic effects of DMT and placebo.

		Placebo	Low infusion (0.6 mg/min)	High infusion (1.0 mg/min)	Low bolus (15 mg) + low infusion (0.6 mg/min)	High bolus (25 mg) + high infusion (1.0 mg/min)	F_4,108_	*p* =
		(mean ± SEM)	(mean ± SEM)	(mean ± SEM)	(mean ± SEM)	(mean ± SEM)		
*Subjective Effects*								
Any drug effect	ΔE_max_	0.5 ± 0.2	5.1 ± 0.5	7.6 ± 0.4	9.4 ± 0.3	10 ± 0	144.4	<0.001
	AUEC	16 ± 12	388 ± 39	623 ± 39	434 ± 43	651 ± 36	50.9	<0.001
	T_max_ (min)	2.6 ± 0.9	27 ± 3.2	28 ± 3.1	2.1 ± 0.1	2.3 ± 0.2	40.6	<0.001
Good drug effect	ΔE_max_	0.6 ± 0.2	5.2 ± 0.5	7.4 ± 0.5	8.3 ± 0.5	9.1 ± 0.3	70.2	<0.001
	AUEC	17 ± 12	378 ± 38	589 ± 43	439 ± 46	588 ± 46	36.2	<0.001
Bad drug effect	ΔE_max_	0 ± 0	0.5 ± 0.2	1.3 ± 0.3	3.5 ± 0.7	4.1 ± 0.7	15.6	<0.001
	AUEC	0.4 ± 0.4	15 ± 5.0	38 ± 12	37 ± 14	69 ± 17	5.2	<0.001
Fear	ΔE_max_	0 ± 0	0.1 ± 0.1	0.1 ± 0.1	1.7 ± 0.5	1.9 ± 0.5	7.5	<0.001
	AUEC	0 ± 0	0.9 ± 0.7	0.6 ± 0.5	4.3 ± 1.6	17 ± 5.5	7.9	<0.001
*Autonomic Effects*								
Systolic blood pressure (mmHg)	E_max_	136 ± 2.9	138 ± 2.2	141 ± 2.1	154 ± 2.9	159 ± 3.4	13.9	<0.001
Diastolic blood pressure (mmHg)	E_max_	84 ± 1.4	87 ± 1	90 ± 1	92 ± 1.5	98 ± 1.6	16.9	<0.001
Heart rate (beats/min)	E_max_	85 ± 3.2	88 ± 2.5	93 ± 3.1	110 ± 3.9	119 ± 4.2	18.5	<0.001
RPP (mmHg x bpm)	E_max_	11320 ± 509	11749 ± 408	12850 ± 468	16950 ± 703	18655 ± 781	31.1	<0.001
Acute adverse effects (LC)	ΔLC score	−0.6 ± 0.4	1.0 ± 0.5	1.9 ± 0.8	2.0 ± 0.8	3.2 ± 0.9	3.9	<0.01
*Hormones*								
BDNF (pg/ml)	Δ90 min	88 ± 191	−107 ± 264	−369 ± 376	−110 ± 302	−288 ± 301	0.4	NS
	Δ150 min	299 ± 229	−285 ± 272	332 ± 633	369 ± 466	205 ± 561	0.3	NS
Oxytocin (pg/ml)	Δ60 min	−11.4 ± 7.6	−4.4 ± 6.9	19 ± 6.0	1.7 ± 6.4	−0.77 ± 5.6	2.9	<0.05

*NS* not significant, *AUEC* Area under the effect curve, *BDNF* Brain-derived neurotrophic factor, *Δ90* *min* the difference from baseline concentration at 90 min, *Δ120* *min* difference from baseline at 120 min, *ΔEmax* maximal effect difference from baseline, *LC* list of complaints, *ΔLC score* the difference of LC score from Baseline, *RPP* Rate pressure product, *Tmax* time to reach Emax, *n* = 27.

### Autonomic and adverse effects

Autonomic effects over time and respective peak effects are shown in Table 1, Supplementary Table S5 and Supplementary Fig. S1. The bolus doses produced a rapid, marked, and short-lasting increase in blood pressure and heart rate that peaked within 2 min (Table 1, Supplementary Fig. S1). DMT infusions only mildly and nonsignificantly elevated blood pressure and heart rate compared with placebo. Vital signs normalized within 15 min of stopping the infusion.

Adverse effects are shown in Table 1, Supplementary Table S2 and S5. Only the high bolus significantly increased adverse effects compared with placebo. Frequent complaints included heart palpitation, nausea, tiredness, uneasiness, and thirst (Supplementary Table S2). The most frequently reported adverse effect after the DMT sessions and that occurred within 48 h after a study session was headache (seven participants, eight times). All episodes of headache were associated with DMT (four times for bolus, four times for infusion). No flashbacks were reported during the study. However, one subject had several flashbacks of moderate to strong intensity after study completion. Flashbacks began 2 weeks after the last study session and lasted for several weeks before resolving completely. There were two severe adverse events during the study (COVID infection with syncope and an elective surgery), which were both unrelated to DMT.

### Plasma oxytocin and BDNF levels

The high infusion increased oxytocin plasma levels, whereas the low infusion and both bolus conditions had no effect compared with placebo. None of the DMT conditions altered plasma BDNF levels compared with placebo (Table 1, Supplementary Table S5, Supplementary Fig. S7+S8).

### Plasma drug concentrations

**Fig. 3:**
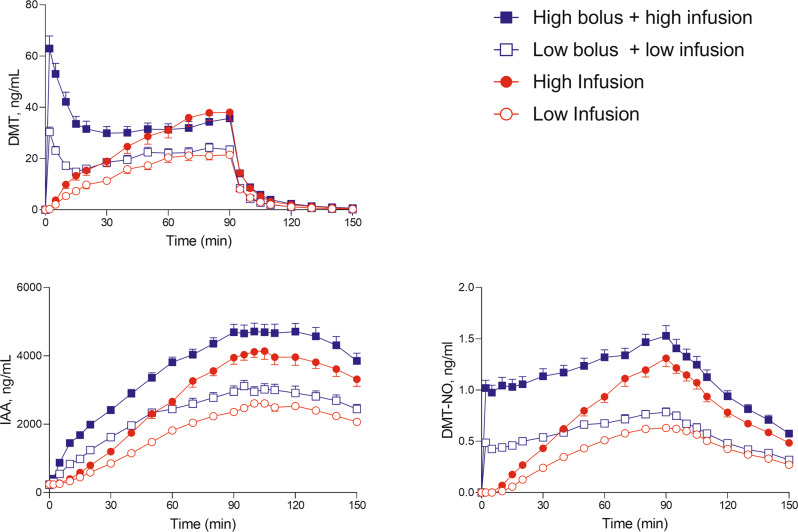
Plasma concentrations of *N,N*-dimethyltryptamine (DMT), DMT-*N*-oxide (DMT-NO), and indole-3-acetic acid (IAA) over time. Both bolus doses (15 and 25 mg) produced peak DMT levels 2 min after administration. Plasma DMT concentrations then rapidly decreased until reaching a plateau after 20 min. In the infusion-only conditions (0.6 and 1 mg/min), DMT concentrations increased until reaching a plateau at 70–80 min. After stopping the infusion at 90 min, DMT levels very rapidly decreased within 15–20 min in all conditions. The corresponding pharmacokinetic parameters are shown in Table 2 and Supplementary Table S4. Individual concentrations are shown in Supplementary Fig. S3–5.

The concentration-time curves for DMT and its metabolites indole-3-acetic acid (IAA) and DMT-*N*-oxide (DMT-NO) are shown in Fig. 3, and individual curves are shown in Supplementary Fig. S2–5. Table 2 and Supplementary Table S4 show the respective pharmacokinetic parameters. The mean maximum plasma concentrations (C_max_) for the 15 and 25 mg DMT bolus doses were 29 and 61 ng/ml, respectively, and were reached at a mean time (T_max_) of 2.5 and 2.9 min, respectively. C_max_ values for the infusion-only conditions were 24 and 39 ng/ml for the low infusion and high infusion, respectively, and were reached (T_max_) after 72 and 73 min, respectively. Two distinct elimination half-lives were observed after stopping the infusion at 90 min: an early elimination half-life (t_1/2α_) with a mean value of 5.0–5.8 min and a late elimination half-life (t_1/2β_) with a mean value of 14–16 min. The switch from early to late elimination occurred 16–18 min after stopping the infusion and at mean DMT concentrations of 2.1–4.2 ng/ml (Supplementary Table S4). Three quarters (75–79%) of the DMT was eliminated during the early elimination phase (Supplementary Table S4). Mean estimates of the apparent volume of distribution (V_z_) were 746–831 L. Mean estimates for clearance were 36–39 l/min (Table 2). The DMT concentration-response relationship over time is shown in Supplementary Fig. S6. We observed moderate acute tolerance to the subjective effects of DMT, evidenced by clockwise hysteresis. Specifically, subjective effects showed no increase from 30 to 90 min, despite increasing plasma concentrations over time (Supplementary Fig. S6). Sex or prior substance experience did not moderate the pharmacokinetic parameters or acute effects of DMT.

**Table 2:** Pharmacokinetic parameters for DMT based on non–compartmental analyses [geometric mean (95% CI), range].

	Low infusion (0.6 mg/min)	High infusion (1.0 mg/min)	Low bolus (15 mg) + low infusion (0.6 mg/min)	High bolus (2 5 mg) + high infusion (1.0 mg/min)
C_max_ (ng/ml)	24 (21–28)	39 (33–45)	29 (26–33)^a^	61 (51–73)^a^
	11–48	15–83	14–56	11–107
T_max_ (min)	72 (67–78)	73 (64–83)	2.5 (2.1–2.9)^a^	2.9 (2.4–3.6)^a^
	40–90	15–90	2–5	2–10
t_1/2α_ (min)	5.3 (4.5–6.1)	5.3 (4.7–5.9)	5.0 (4.4–5.6)	5.8 (5.1–6.5)
	3.0–15	3.2–10.0	2.9–11	2.8–11
t_1/2β_ (min)	15 (13–16)	15 (14–16)	14 (13–16)	16 (14–17)
	7.8–29	10–22	7.5–23	10–22
CL (L/min)	39 (34–46)	39 (33–46)	36 (32–42)	36 (32–40)
	16–97	16–91	21–82	22–61
V_z_ (L)	820 (671–1004)	831 (683–1012)	746 (639–871)	799 (693–922)
	287–2765	284–2133	388–1945	379–1752
AUC_∞_ (ng^a^min/ml)	1374 (1174–1607)	2303 (1966–2697)	1902 (1653–2189)	3220 (2889–3588)
	554–3374	987–5764	839–3212	1883–5247

*AUC* area under the plasma concentration-time curve, *AUC*_*∞*_ AUC from time zero to infinity, *CL* apparent total clearance, *C*_*max*_ maximum observed plasma concentration, *t*_*1/2α*_ early elimination plasma half-life, *t*_*1/2β*_ late elimination plasma half-life, *T*_*max*_ time to reach C_max,_ *95%CI* 95% confidence interval, *V*_*z*_ apparent volume of distribution, *n* = 27.

^a^In the DMT bolus conditions, Cmax and Tmax values were calculated considering only plasma concentrations within the first 15 min after the bolus application.

### Blinding

Data on the participants’ retrospective identification of the substance and dose are shown in Supplementary Table S3. Overall, most conditions were correctly identified. However, the low infusion-only condition was mistaken for either the placebo (7%) or high infusion-only (26%) condition after the study session. The low bolus + low infusion condition was misclassified as the high bolus + high infusion (19%) or placebo (4%) condition after the session.

## Discussion

The present study characterized pharmacokinetics and pharmacodynamics of DMT at low and high doses that were administered via 90-min continuous intravenous infusions with and without an initial loading bolus. The bolus dose was moderately well tolerated and typically described by the participants as a very intense and overwhelming experience that was perceived simultaneously as good, bad and anxiety-inducing. After the bolus administration of DMT at doses of 15 and 25 mg, plasma DMT concentrations peaked at 2.5 and 2.9 min and rapidly declined over 15 min. Subjective effects of DMT developed very rapidly during the 45-s bolus administration, reached a maximum within 2–3 min, and also decreased within approximately 15 min. In contrast, DMT administration as a continuous infusion at a stable rate of 0.6 or 1 mg/min over 90 min produced slowly rising plasma DMT concentrations, with maximal concentrations within 72 min. Subjective effects after DMT administration as an infusion increased slowly and gradually over 20–30 min when a plateau was reached until the infusion was ended at 90 min. The bolus administrations produced significantly greater subjective effects than the infusions. This was also the case for the low bolus dose compared with the high infusion. Thus, although the high infusion resulted in higher plasma DMT concentrations than the low bolus dose, the subjective effects of the low bolus were more intense. This is likely attributable to the more rapid rate of increase in DMT concentrations and more rapid onset of subjective effects during a DMT bolus vs. infusion. Thus, the participants considered the effects of DMT less intense when plasma levels of DMT rose more slowly compared with a rapid increase that was produced by the bolus administration. Possibly, the bolus doses produced high initial brain concentrations by rapid uptake of DMT at the blood-brain barrier followed by a slower washout and gradual distribution to other compartments. The bolus administrations were associated with significantly greater “bad drug effects”, anxiety, and cardiovascular stimulant effects at the onset compared with infusions of DMT. Similarly, different doses of 7–20 mg DMT that were administered in 13 participants produced high subjective peak effects within 1–3 min, which disappeared within 20 min [5, 18, 19]. Similar subjective effects were also described after the administration of intravenous DMT bolus doses of 0.2 and 0.4 mg/kg (~15 and 30 mg, respectively) in previous studies [8, 9]. At the 0.4 mg/kg dose, participants reportedly were almost uniformly overwhelmed by the intensity and speed of onset of effects of this dose [8, 9]. All participants reportedly described an intense, rapidly developing, and usually transiently anxiety-provoking “rush” throughout the body and mind [8, 9]. Most participants lost awareness of their bodies, and many were not cognizant of being in the hospital or participating in an experiment for the first minute or two of the experience [8]. Thus, we consider that the bolus doses that were used in our study were high, and/or the durations of administration were too short. The administration of DMT in 20 ml of saline further enhanced the flush-in and rapid access to the circulation compared with the administration in 1 ml reported by others [10]. Overall, a rapid onset may likely not be beneficial, and slower induction by using, for example, 1–4 mg DMT/min over 5–10 min may be better tolerated and have a more gradual onset while still reaching an intense state. Alternatively, continuous infusion schedules like the one used in the present study are well-tolerated and result in shorter treatment times compared to orally administered psychedelics. Overall DMT induced qualitatively and quantitatively similar psychedelic and mystical effects compared with LSD and psilocybin in a comparable setting with numerically slightly higher ratings on disembodiment at the high bolus dose [22]. The bolus–infusion combination that was used in the present study essentially tested a DMT administration regimen as theoretically proposed in the pharmacokinetic/pharmacodynamic model of Gallimore and Strassman [4]. However, although the suggested high bolus dose (25 mg) and infusion rate (1 mg/min) were used, we did not use the higher infusion rate (3.3 mg/min when starting the infusion) as proposed [4]. In the present study, this resulted in only a minimal drop in subjective effects after the bolus and before the plateau was reached. Additionally, the theoretical model predicted that although plasma concentrations would spike high, as confirmed in our study, the desired effect site concentrations would be reached smoothly with very little overshoot. However, in the present study, subjective effects of the bolus dose were greater than the predicted effects in the model, indicating that DMT reached the brain more rapidly and also resulted in higher plasma concentrations and effects than theoretically predicted [4]. Additionally, we documented stable subjective effects of DMT within 30 min of the start of the infusion and up to 90 min, despite further increasing plasma DMT concentrations within this time period and up to 73 min, consistent with moderate acute pharmacological tolerance to continuous DMT administration. This finding is also illustrated by clockwise hysteresis in the DMT effect–concentration curve over time. This acute tolerance during continuous administration via infusion contrasted with the reported absence of tolerance for retrospectively reported subjective effects with repeated bolus injections [6]. Thus, tolerance to DMT effects may be observed with continuous but not with intermittent very short-lasting administrations when DMT concentrations and effects are decreasing between administrations. Further studies with longer infusion times are needed to confirm this observation.

The present study also validly determined pharmacokinetic parameters of DMT using concentration measurements after stopping the infusion. A few previous studies measured DMT concentrations after bolus administrations not allowing to distinguish between DMT distribution and elimination [2, 8, 11, 18]. The present larger pharmacokinetic study showed that 75–79% of DMT was very rapidly cleared from plasma, with an initial plasma half-life of 5–6 min over 16–17 min until plasma DMT concentration of 2–4 ng/ml were reached. The plasma DMT concentration then further decreased more slowly, with a late plasma half-life of 15 min. Subjective effects declined in parallel with the rapid half-life within 15 min. The rapid initial elimination reflects the MAO activity and rapid metabolism of DMT. The observed DMT clearance was also very high (36–39 l/min) in the present study and similar to the previously reported 26 l/min in 13 participants [18]. Essentially, the observation of two distinct elimination half-lives suggests the presence of distribution kinetics. As such, the late elimination phase may reflect the rate-limiting redistribution of DMT from tissue and the intracellular space back into the circulation and not elimination. These considerations are important for the conceptualization of possible compartmental models for the pharmacokinetics of DMT, which we did not establish for the present publication. Evidence of distributional kinetics was also previously reported in a pharmacokinetic pilot study that used a two-compartment model to describe the kinetics of DMT, essentially implying the presence of an additional distributional component [18]. However, the possible redistribution of DMT becomes apparent at very low plasma levels, when most subjective effects have already vanished.

DMT showed no consistent effects on neuroendocrine markers such as oxytocin and BDNF. Only the high infusion of DMT increased circulating oxytocin compared with placebo. However, sampling at 60 min may have been too late to detect oxytocin changes after the bolus dose. Plasma BDNF levels were not altered by DMT. Some previous studies found increases in plasma or serum BDNF levels in response to psychedelics [23, 25, 37], whereas some did not [22]. Thus, further studies are needed to confirm the endocrine effects of DMT.

The present study has several strengths. We used a relatively large study sample (*n* = 27) with 108 DMT administrations and powerful within-subject comparisons in a randomized double-blind design. DMT doses were pharmacologically well-defined. We included equal numbers of male and female participants and used internationally established psychometric outcome measures. Plasma DMT concentrations were determined at close intervals in all participants and analyzed with a validated analytical method. Pharmacokinetics of DMT were characterized after stopping the infusion and in a large sample, thus allowing the valid determination of elimination parameters including inter-individual variation. Notwithstanding these strengths, the present study also has limitations. The study used a highly controlled hospital setting and included only healthy volunteers. Thus, subjects in different environments and patients with psychiatric disorders may respond differently to DMT. Moreover, most participants correctly guessed the order of treatment allocation after the completion of the study and thus treatments were unblinded in most cases. However, the psychedelic experience itself and in particular the very intense bolus effects are almost by definition unblinding. As such, other designs such as the use of an active placebo comparator would have been unlikely to meaningfully increase blinding rates.

## Conclusion

We characterized effects of different intravenous DMT administration regimens to support dose finding for research and psychedelic-assisted therapy in future studies. DMT produced rapid and intense short-lasting and moderately well-tolerated psychedelic effects when dosed as a bolus, and it induced stable, intense, and well-tolerated effects when used as a infusion. Intravenous DMT administration, particularly as an infusion, may be used as a pharmacological tool to rapidly induce, maintain, and end a psychedelic state in a highly controlled manner. Thus, a psychedelic experience can be tailored to the specific needs of patients and therapeutic settings.

## Supplementary information

Supplement
