# Supplementary material for: Acute effects of intravenous DMT in a randomized placebo-controlled study in healthy participants
Source: Transl Psychiatry. 2023 May 23;13:172. doi: 10.1038/s41398-023-02477-4 (PMC10206108; doi:10.1038/s41398-023-02477-4)
Supplement: Supplementary file 1 — Supplement [file 41398_2023_2477_MOESM1_ESM.pdf]

Supplement

Methods

Subjective effect scales (SES)

Participants were asked by the investigator to repeatedly rate their subjective effects verbally on a Likert scale from 0 to 10 for: “any drug effect”, “good drug effect”, “bad drug effect”, and “fear”. Intervals were 1 h before and 0, 2, 3.5, 5, 7.5, 10, 12.5, 15, 20, 30, 40, 50, 60 70, 80, 90, 92.5, 95,100, 105, 110, 120, 130, 140 and 150 minutes after drug administration. A similar method was used previously to assess DMT effect [1] and is less demanding than completing self-rated VAS in written form [2] and therefore interferes only minimally with the subjective experience. The SES “any drug effect” is an overall effect measure to characterize the overall effect intensity and time course. The SES “good drug effect” is an overall measure of effects subjectively considered positive. The SES “bad drug effect” is an overall measure of any negative effects and related to “fear”. SES scales were assessed each time DMT blood concentrations were measured to allow for pharmacokinetic-pharmacodynamic modeling.

5 Dimensions of Altered States of Consciousness (5D-ASC) scale

The 5 Dimensions of Altered States of Consciousness (5D-ASC) scale [3, 4] was used as the primary outcome measure and was administered 150 minutes after drug administration to retrospectively rate peak drug effects. The 5D-ASC scale measures altered states of consciousness and contains 94 items (visual analog scales). The instrument consists of five subscales/dimensions [3] and 11 lower-order scales [4]. The 5D-ASC dimension “Oceanic Boundlessness” (27 items) measures derealization and depersonalization associated with positive emotional states, ranging from heightened mood to euphoric exaltation. The corresponding lower-order scales include “experience of unity,” “spiritual experience,” “blissful state,” “insightfulness,” and “disembodiment.” The dimension “Anxious Ego Dissolution” (21 items) summarizes ego-disintegration and loss of self-control phenomena associated with anxiety. The corresponding lower-order scales include “impaired control of cognition” and “anxiety.” The dimension “Visionary Restructuralization” (18 items) consists of the lower-order scales “complex imagery,” “elementary imagery,” “audio-visual synesthesia,” and “changed meaning of percepts.” Two additional dimensions describe “Auditory Alterations” (15 items) and “Reduction of Vigilance” (12 items). The total 3D-ASC score is the total of the three main dimensions “Oceanic Boundlessness”, “Anxious Ego-Dissolution”, and “Visionary Restructuralization” and can be used as a measure of the overall intensity of the alteration of the mind [5]. The scale is well-validated in German [3] and many other languages and widely used to characterize the subjective effects of various psychedelic drugs. In particular, the scale

has been used by most research groups to psychometrically assess LSD effects [6-11]. Furthermore, acute ratings on the 5D-ASC after administration of psilocybin have been used to predict long-term effects of psychedelic treatments in patients [12, 13]. Ratings on the 5D-ASC have been shown to closely correlate with ratings on the Mystical Effects Questionnaire (MEQ, see below) [5] which is primarily used by research groups in the US [13].

Mystical Effects Questionnaire (MEQ30)

Mystical experiences were assessed 150 minutes after drug administration using the 100-item States of Consciousness Questionnaire (SOCQ) [5, 14] that includes the 43-item Mystical Effects Questionnaire (MEQ43) [14], 30-item Mystical Effects Questionnaire (MEQ30) [15], and subscales for “aesthetic experience” and negative “nadir” effects. The published German version was used [5]. The MEQ has been used in numerous experimental and therapeutic trials with psilocybin [13, 14, 16-22]. The MEQ items provide scale scores for each of seven domains of mystical experiences: internal unity, external unity, sacredness, noetic quality (as real as or more real than everyday reality), deeply felt positive mood, transcendence of time and space, and ineffability/paradoxicality (difficulty describing the experience in words). The total of all scale scores was used as an overall measure of the mystical-type experience. We also derived the four scale scores of the newly validated revised 30-item MEQ: mystical, positive mood, transcendence of time and space, and ineffability [15]. A complete mystical experience was defined as scores ≥ 60% on all MEQ30 factors [15]. For the scale validation see [15]. For the German translation of the MEQ30 see online supplement of [5].

Results

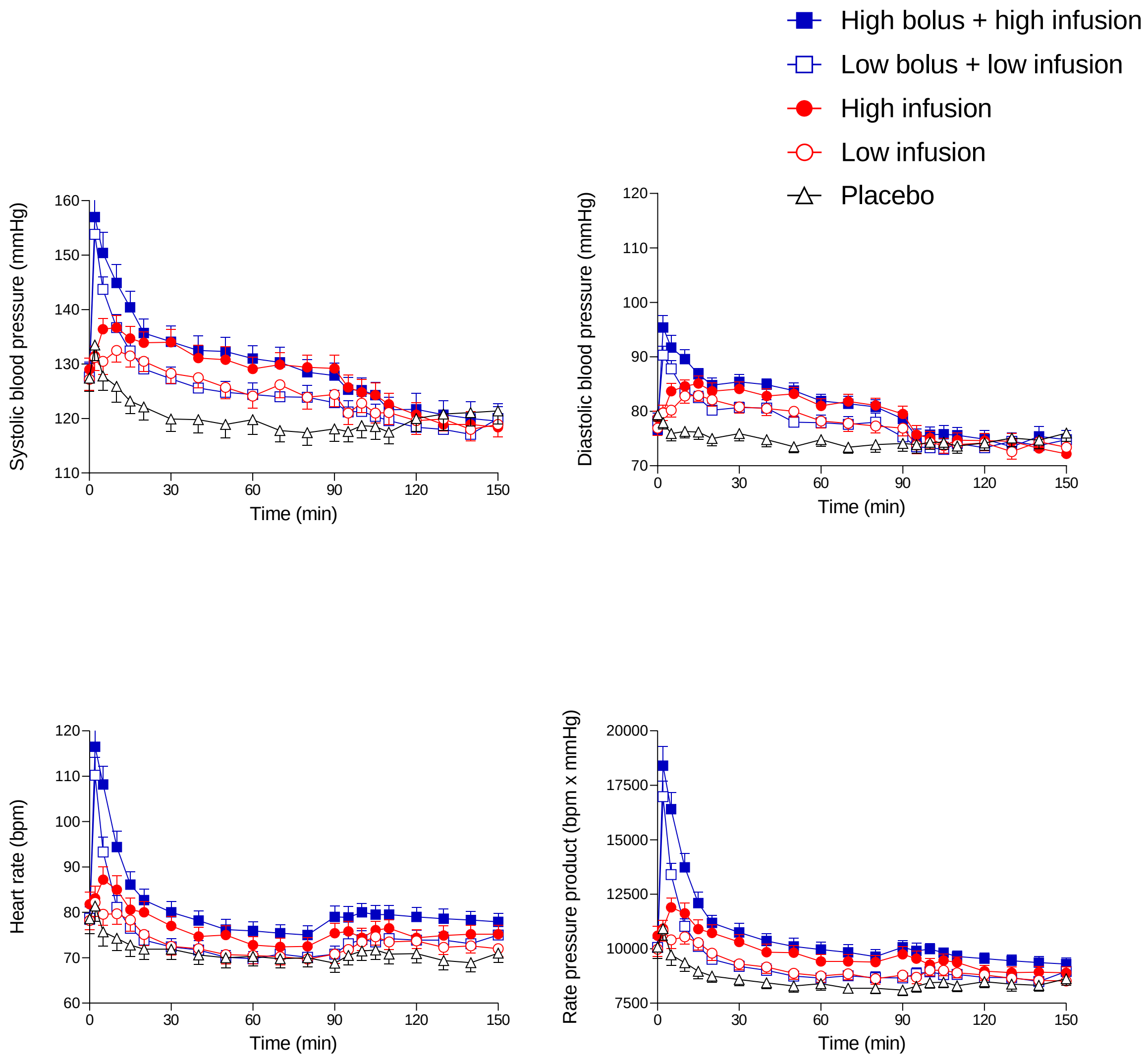

Figure S1. Acute autonomic effects of *N,N*-dimethyltryptamine (DMT) over time. Both the low (15 mg) and high (25 mg) DMT bolus doses rapidly and markedly elevated blood pressure and heart rate, which peaked within the first 2 min after intravenous bolus administration. Infusions moderately increased blood pressure compared with placebo, and effects normalized within 15 min of stopping the infusion. The data are expressed as the mean ± SEM in 27 subjects. Maximal effects and statistics are shown in Table 1 and Table S5.

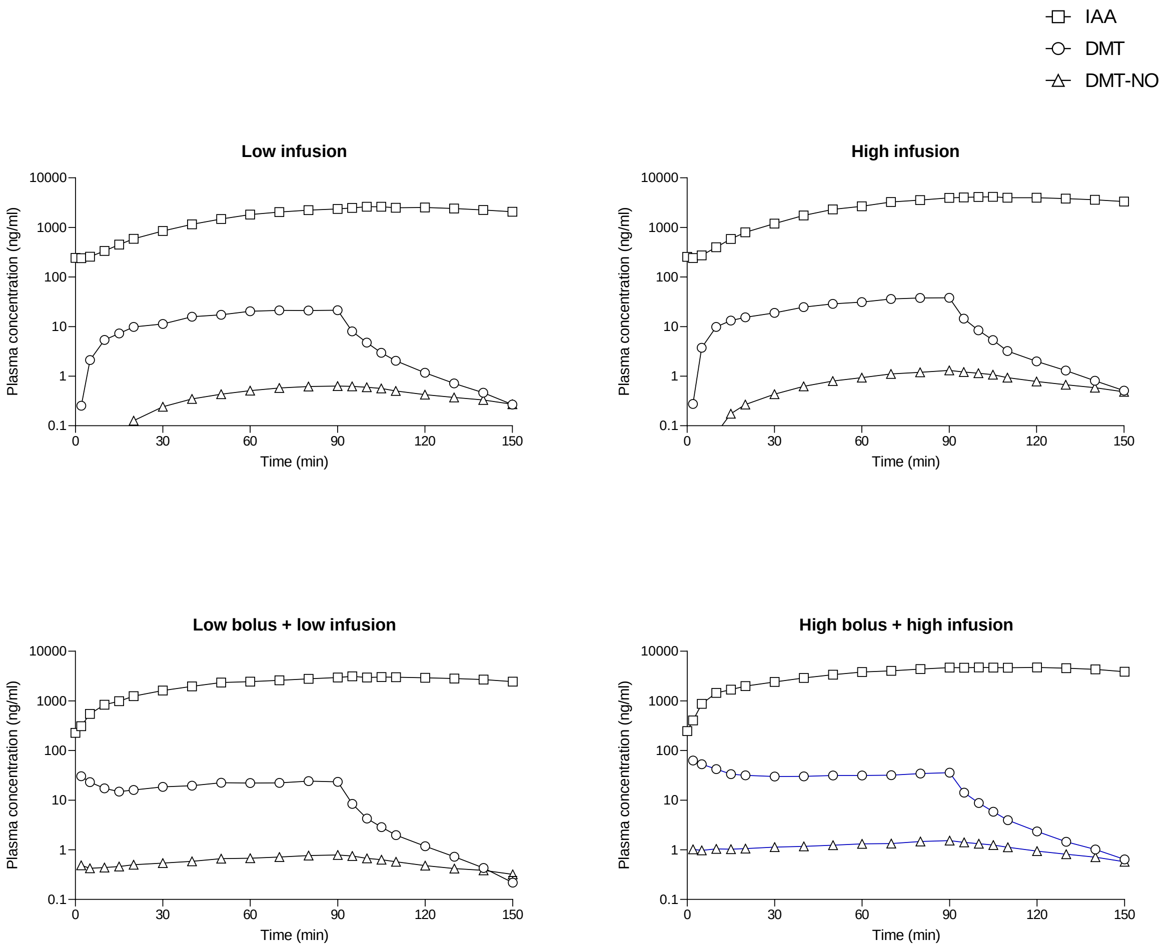

Figure S2. Plasma concentrations of *N,N*-dimethyltryptamine (DMT), DMT-*N*-oxide (DMT-NO), and indole-3-acetic acid (IAA) over time on semilogarithmic plots. There was an early fast elimination, with a half-life ($t_{1/2\alpha}$) of approximately 5.4 min during the first 17 min after stopping the infusion at 90 min, followed by late slower decline with a half-life ($t_{1/2\beta}$) of 15 min (Table 2, Supplementary Table S4). The data are expressed as means. SEMs are not displayed because of the logarithmic scale on the y-axis.

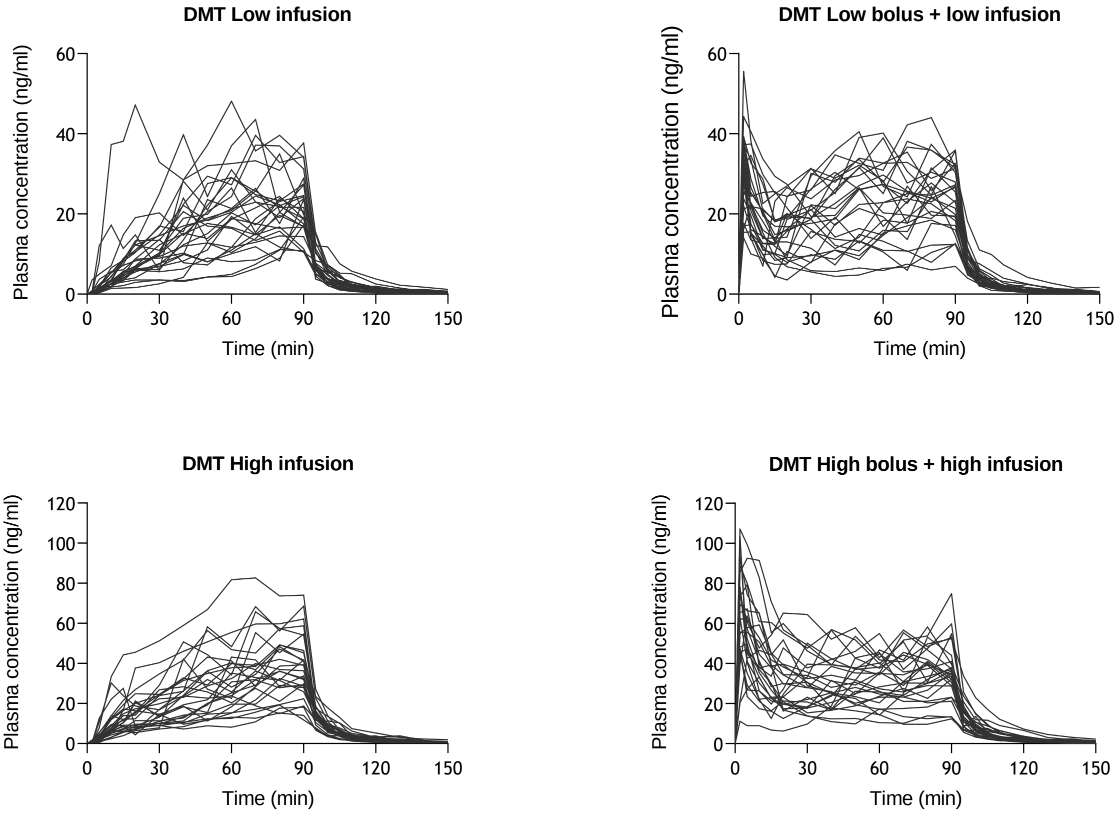

Figure S3. Plasma concentrations of *N,N*-dimethyltryptamine (DMT) over time. Each line represents a single subject (*n* = 27). Low (15 mg) and high (25 mg) bolus doses were administered at t = 0. Low (0.6 mg/min) and high (1 mg/min) infusions started at t = 1 and lasted until t = 90 min.

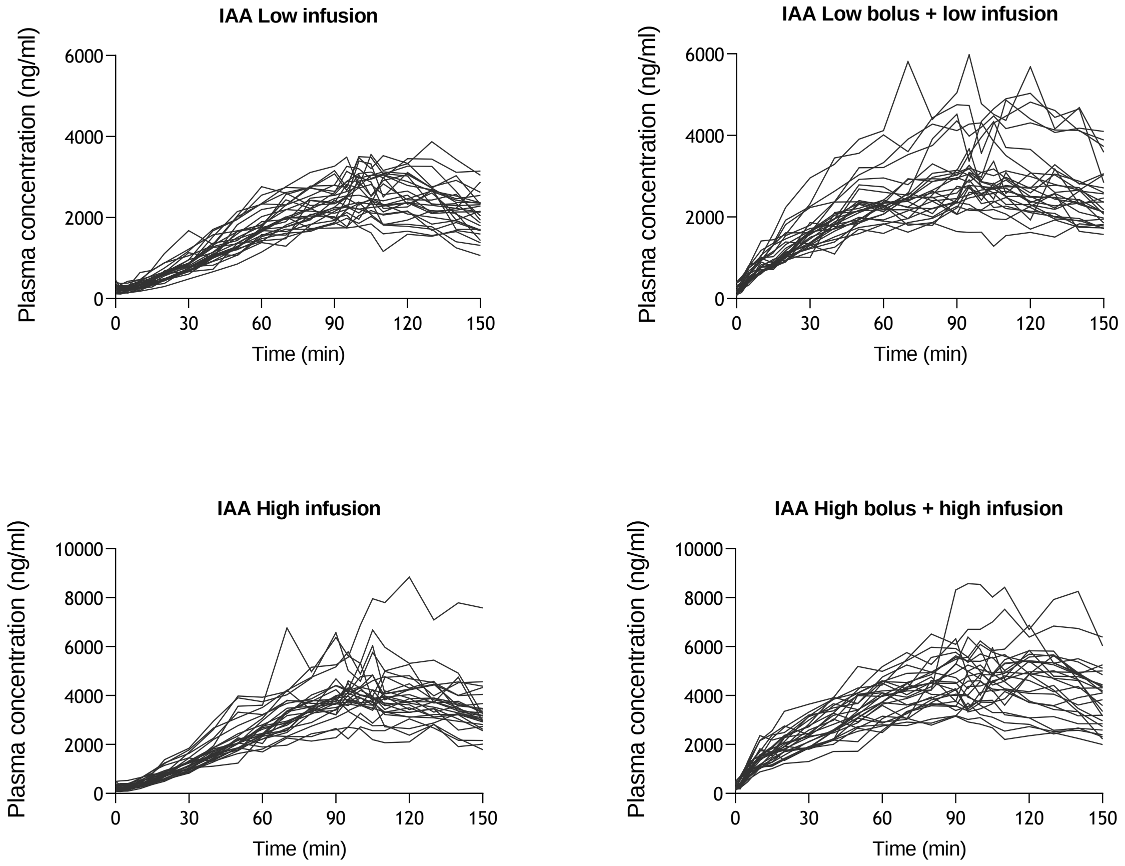

Figure S4. Plasma concentrations of indole-3-acetic acid (IAA) over time. Each line represents a single subject (total *n* = 27). Low (15 mg) and high (25 mg) bolus doses were administered at t = 0. Low (0.6 mg/min) and high (1 mg/min) infusions started at t = 1 and lasted until t = 90 min.

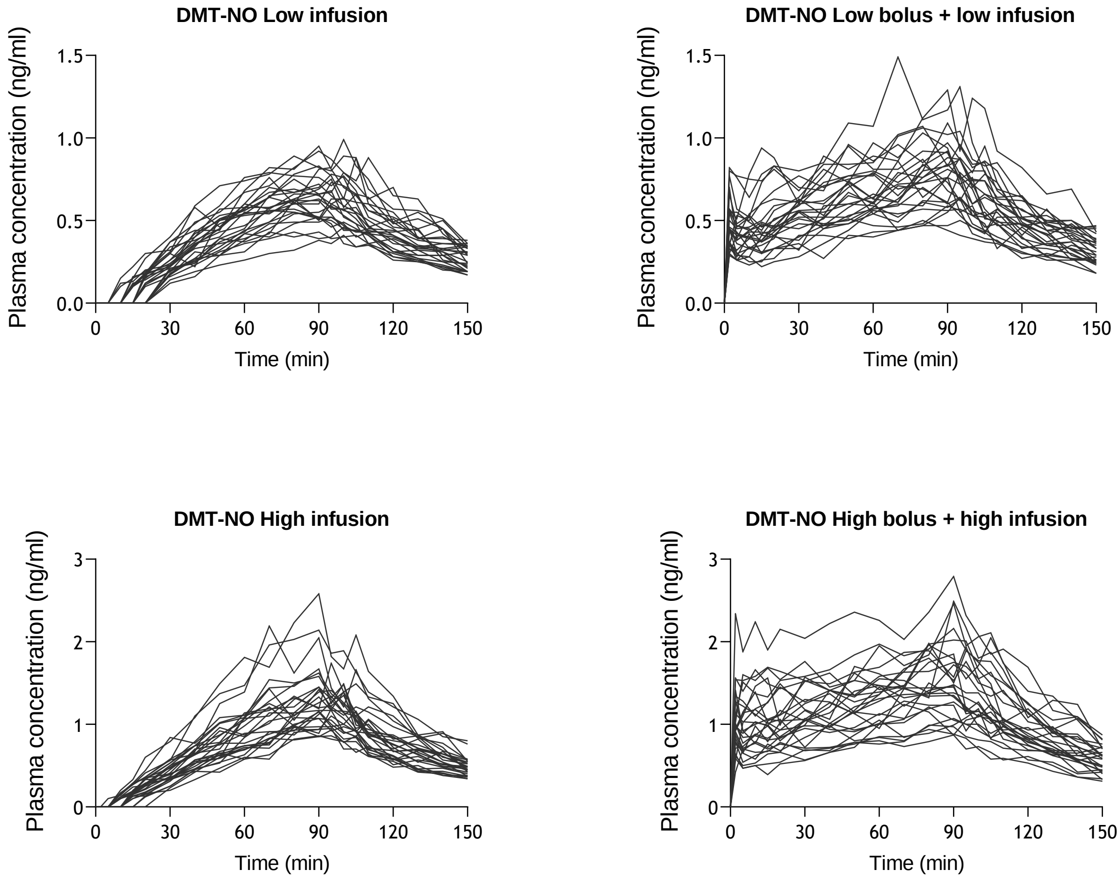

Figure S5. Plasma concentrations of DMT-*N*-oxide (DMT-NO) over time. Each line represents a single subject (*n* = 27). Low (15 mg) and high (25 mg) bolus doses were administered at t = 0. Low (0.6 mg/min) and high (1 mg/min) infusions started at t = 1 and lasted until t = 90 min.

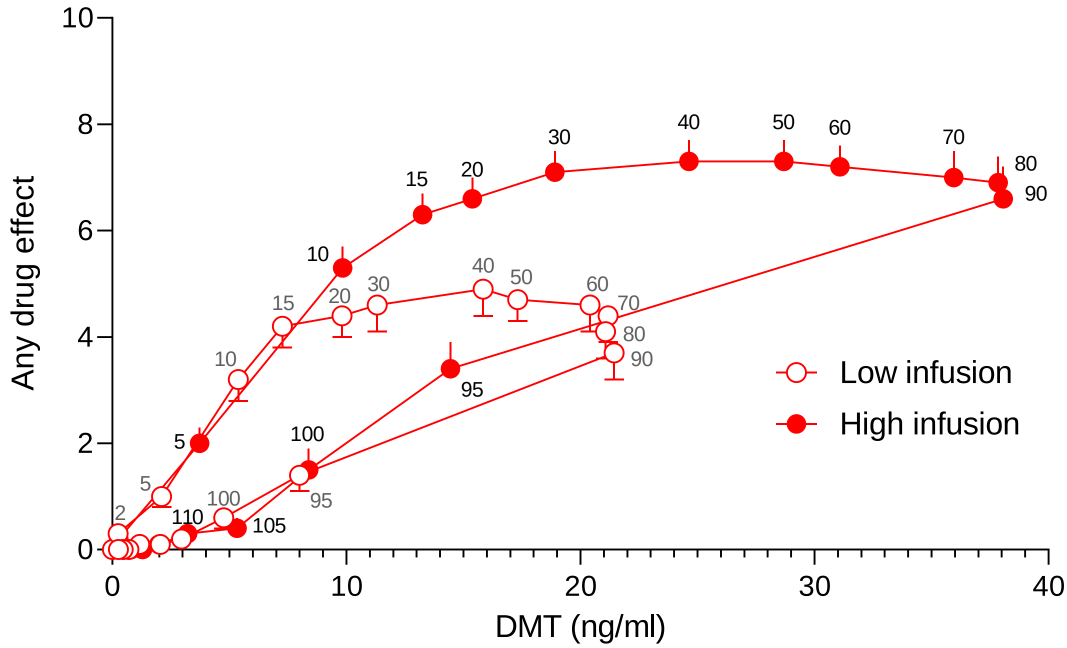

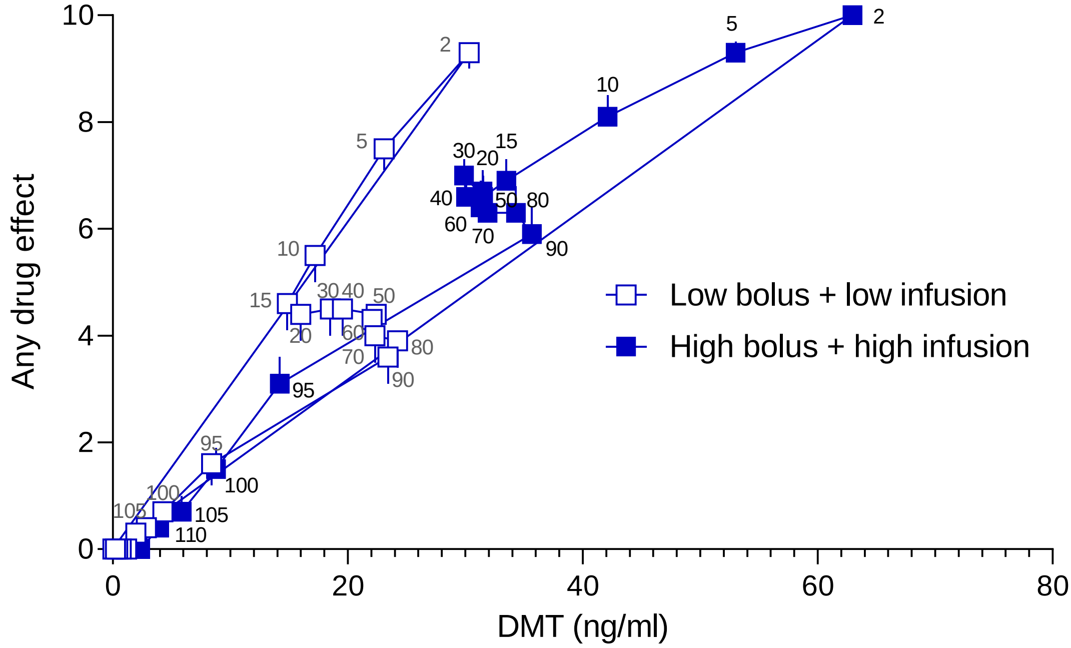

Figure S6. Plasma concentration-effect relationship of DMT. Plasma concentration values are geometric means, and effects are mean ± SEM scores on the subjective effects scale (SES) of "any drug effect." The time of sampling (in minutes) is indicated next to each data point. There was moderate clockwise hysteresis in the infusions conditions, indicating acute tolerance.

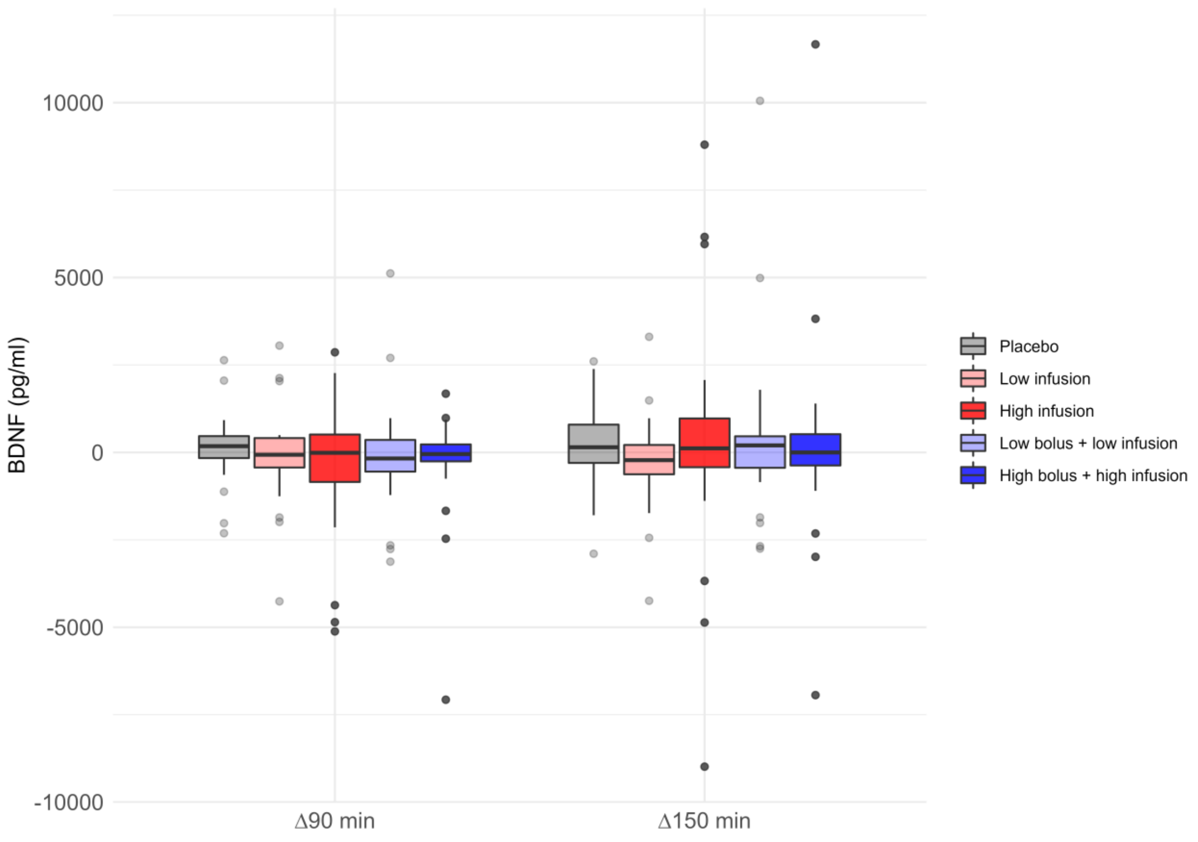

Figure S7. Plasma concentrations of brain-derived neurotrophic factor (BDNF), showing the difference from baseline (t = 0) at 90 and 150 min. The corresponding statistics are shown in Table 1 and Table S5.

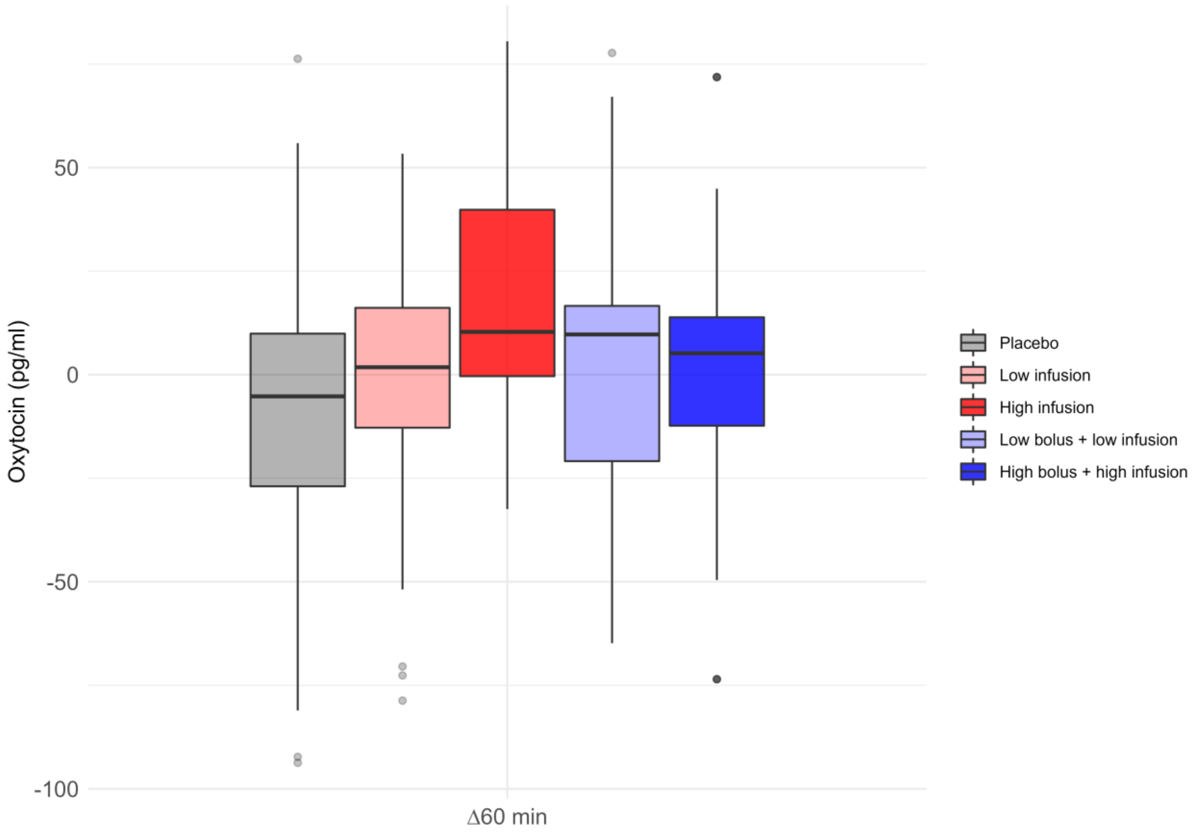

Figure S8. Plasma concentrations of Oxytocin at baseline (0 min) and at 60 min. The corresponding statistics are shown in Table 1 and Table S5.

Table S1. Mean values and statistics for the acute subjective effects of the four DMT conditions and Placebo on the 5 Dimensions of Altered States of Consciousness (5D-ASC) Scale and the Mystical Experiences Questionnaire (MEQ)

		Placebo	Low infusion (0.6 mg/min)	High infusion (1.0 mg/min)	Low bolus (15 mg) + low infusion (0.6 mg/min)	High bolus (25 mg) + high infusion (1.0 mg/min)	$F_{4,108}$	*p* =	Pla vs. LI	Pla vs. HI	Pla vs. LB + LI	Pla vs. HB + HI	LI vs. HI	LI vs. LB + LI	LI vs. HB + HI	HI vs. LB + LI	HI vs. HB + HI	LB + LI vs. HB + HI
		(mean ± SEM)	(mean ± SEM)	(mean ± SEM)	(mean ± SEM)	(mean ± SEM)												
5 Dimensions of Altered States of Consciousness (5D-ASC) Scale																		
5D-ASC total Score	% score	0.4 ± 0.2	18 ± 2.9	23 ± 2.8	23 ± 2.9	31 ± 2.3	21.6	< 0.001	***	***	***	***	NS	NS	**	NS	NS	NS
3D-ASC total Score	% score	0.1 ± 0.1	20 ± 3.2	26 ± 3.2	26 ± 3.1	35 ± 2.5	23.8	< 0.001	***	***	***	***	NS	NS	***	NS	NS	NS
Oceanic boundlessness	% score	0.2 ± 0.1	26 ± 4.5	34 ± 4.6	33 ± 4.3	43 ± 3.9	17	< 0.001	***	***	***	***	NS	NS	*	NS	NS	NS
Anxious ego-dissolution	% score	0 ± 0	5.8 ± 1.8	7.9 ± 1.5	13 ± 2.7	20 ± 2.8	17.6	< 0.001	NS	*	***	***	NS	*	***	NS	***	*
Visionary restructuralization	% score	0.2 ± 0.1	27 ± 4.4	38 ± 4.7	33 ± 3.8	43 ± 3.1	20.3	< 0.001	***	***	***	***	NS	NS	NS	NS	NS	NS
Auditory alterations	% score	0 ± 0	5.7 ± 2.5	6.7 ± 2.3	7.3 ± 2.2	10 ± 3.1	2.6	< 0.05	NS	NS	NS	*	NS	NS	NS	NS	NS	NS
Reductions of vigilance	% score	2.6 ± 1.1	21 ± 4.2	21 ± 4.2	24 ± 4.1	32 ± 4.4	8.2	< 0.001	**	**	***	***	NS	NS	NS	NS	NS	NS
Experience of unity	% score	0.1 ± 0.1	22± 4.8	29 ± 4.8	27 ± 5.2	36 ± 6.0	8.6	< 0.001	**	***	***	***	NS	NS	NS	NS	NS	NS
Spiritual experience	% score	0 ± 0	15 ± 4.7	22 ± 5.0	19 ± 5.3	26 ± 5.3	4.9	< 0.01	NS	**	*	***	NS	NS	NS	NS	NS	NS
Blissful state	% score	0.2 ± 0.1	41 ± 5.8	48 ± 5.6	36 ± 5.2	48 ± 5.9	15.5	< 0.001	***	***	***	***	NS	NS	NS	NS	NS	NS
Insightfulness	% score	0.5 ± 0.4	17 ± 3.5	26 ± 5.0	25 ± 5.0	28 ± 4.6	7.8	< 0.001	*	***	***	***	NS	NS	NS	NS	NS	NS
Disembodiment	% score	0.2 ± 0.2	23 ± 5.6	30 ± 6.4	43 ± 6.2	50 ± 6.5	12.2	< 0.001	*	**	***	***	NS	NS	**	NS	NS	NS
Impaired control and cognition	% score	0 ± 0	8.3 ± 2.0	14 ± 2.8	20 ± 4.3	29 ± 4.4	11.9	< 0.001	NS	*	***	***	NS	NS	***	NS	*	NS
Anxiety	% score	0 ± 0	0.3 ± 0.1	1.6 ± 0.6	6.9 ± 2.6	13 ± 3.1	9.8	< 0.001	NS	NS	NS	***	NS	NS	***	NS	***	NS
Complex imagery	% score	0.6 ± 0.5	27 ± 5.2	41 ± 5.8	35 ± 5.5	40 ± 5.4	11.5	< 0.001	**	***	***	***	NS	NS	NS	NS	NS	NS
Elementary imagery	% score	0.4 ± 0.3	44 ± 7.1	59 ± 6.6	55 ± 6.0	69 ± 5.5	22.4	< 0.001	***	***	***	***	NS	NS	*	NS	NS	NS
Audio-visual synesthesia	% score	0.1 ± 0.1	46 ± 7.4	53 ± 7.3	42 ± 7.3	53 ± 6.7	11.9	< 0.001	***	***	***	***	NS	NS	NS	NS	NS	NS
Changed meaning of percepts	% score	0.1 ± 0.1	19 ± 3.7	22 ± 5.0	15 ± 3.7	26 ± 4.6	6.7	< 0.001	**	**	*	***	NS	NS	NS	NS	NS	NS
Mystical Experiences Questionnaire (MEQ)																		
<u>MEQ30</u>																		
Mystical	% score	0.2 ± 0.2	19 ± 3.9	22 ± 3.7	19 ± 3.3	26 ± 3.9	9	< 0.001	***	***	**	***	NS	NS	NS	NS	NS	NS
Positive mood	% score	2.1 ± 1.1	35 ± 4.1	40 ± 4.2	39± 3.7	45 ± 3.8	23.2	< 0.001	***	***	***	***	NS	NS	NS	NS	NS	NS
Transcendence of time/space	% score	0.7 ± 0.5	20 ± 4.1	31 ± 4.1	37 ± 4.3	53 ± 4.2	27.1	< 0.001	**	***	***	***	NS	*	***	NS	***	*
Ineffability	% score	0.4 ± 0.4	39 ± 5.6	51 ± 4.9	56 ± 4.7	70 ± 2.7	41.3	< 0.001	***	***	***	***	NS	*	***	NS	*	NS
Total score	% score	0.9 ± 0.5	28 ± 3.8	36 ± 3.7	38 ± 3.2	49 ± 2.7	34.9	< 0.001	***	***	***	***	NS	NS	***	NS	*	NS
Nadir effects	% score	0.3 ± 0.2	4.0 ± 1.0	7.5 ± 1.3	10 ± 1.6	16 ± 2.0	20.1	< 0.001	NS	**	***	***	NS	*	***	NS	***	*
Aesthetic Experience	% score	0.7 ± 0.4	26 ± 2.7	35 ± 3.0	31 ± 3.2	39 ± 3.2	30.7	< 0.001	***	***	***	***	NS	NS	*	NS	NS	NS
<u>MEQ43</u>																		
Internal unity	% score	0.5 ± 0.5	19 ± 4	22 ± 3.9	21 ± 3.4	31 ± 3.7	10.7	< 0.001	**	***	***	***	NS	NS	NS	NS	NS	NS
External unity	% score	0.1 ± 0.1	15 ± 3.4	16 ± 3.2	14 ± 2.9	21 ± 3.4	7.5	< 0.001	**	**	**	***	NS	NS	NS	NS	NS	NS
Sacredness	% score	0.4 ± 0.3	23 ± 3.9	28 ± 4.5	26 ± 3.7	34 ± 3.9	13.1	< 0.001	***	***	***	***	NS	NS	NS	NS	NS	NS
Noetic quality	% score	0.5 ± 0.5	21 ± 4	24 ± 4.1	20 ± 3.6	28 ± 4.3	8.6	< 0.001	***	***	**	***	NS	NS	NS	NS	NS	NS
Positive mood	% score	1.5 ± 0.8	35 ± 4.1	40 ± 4.2	36 ± 3.8	41 ± 4.3	19.9	< 0.001	***	***	***	***	NS	NS	NS	NS	NS	NS
Transcendence of time/space	% score	0.7 ± 0.5	20 ± 4.2	31 ± 4.2	35 ± 4.1	50 ± 4.3	23.9	< 0.001	**	***	***	***	NS	*	***	NS	**	NS
Ineffability	% score	0.2 ± 0.2	28 ± 4.2	38 ± 3.9	41 ± 3.9	51 ± 2.9	33.6	< 0.001	***	***	***	***	NS	NS	***	NS	*	NS

*P<0.05, **P<0.01, ***P<0.001; NS, not significant; HB, High bolus; HI, High infusion; LB, Low bolus; LI, Low infusion; *n* = 27.

Table S2 Acute adverse drug effects

Complaints	Placebo		Low infusion		High infusion		Low bolus + low infusion		High bolus + high infusion	
	0 min	0-150 min	0 min	0-150 min	0 min	0-150 min	0 min	0-150 min	0 min	0-150 min
Heart Palpitation	0	0	0	4	2	5	2	4	0	6
Feeling Of Weekness	1	1	0	3	1	4	1	4	1	4
Nausea	0	0	0	1	0	3	0	3	0	4
Restless Legs	0	0	0	2	0	3	0	4	0	2
Thirst	2	0	1	3	1	5	1	3	1	6
To Cry Easily	1	0	0	3	1	2	0	2	0	6
Headache	2	3	1	6	3	5	3	3	3	5
Lack Of Energy	1	3	1	3	2	3	2	6	2	2
Tiredness	6	5	8	7	11	11	2	7	8	13
Lack Of Concentration	1	1	0	0	0	2	0	3	1	4
Hypersensitivity To Cold	1	1	1	4	0	1	1	2	2	5
Hot Flushs	0	0	0	1	0	3	0	3	1	2
Tremor	0	0	0	1	0	3	0	2	0	2
Easily Exhausted	1	2	1	2	1	3	0	3	2	2
Nightmarish	1	0	0	1	0	2	0	2	0	3
Feeling Of Having A Lump In The Throat	0	0	2	1	1	4	1	3	0	3
Numbness In Hands And Feet	0	0	0	2	0	0	0	2	0	3
Shortag In Breath	0	0	0	0	0	1	0	2	0	4
Lassitude	2	2	1	1	0	2	1	2	1	4
Addephagia	0	1	0	1	0	1	0	1	0	2
Uneasiness	2	0	2	1	1	5	4	4	2	6
Lack Of Appetite	1	0	0	1	2	3	0	2	0	1
Musing	2	1	0	1	0	1	2	3	1	3
Hypersensitivity To Warm	0	0	0	1	0	0	0	1	0	2
Forgetful	1	1	0	1	0	1	1	3	1	1
Sweating	3	0	0	2	0	2	1	1	0	2
Vertigo	1	0	0	1	0	2	1	0	0	2
Feelings Of Fear	1	0	0	0	1	0	2	3	1	4
Feeling Of Satiety	0	0	1	0	0	2	0	1	0	0
Need Of Sleep	1	1	0	1	1	1	0	1	1	1
Difficulties To Breath	0	0	0	0	0	1	0	0	0	1
Freeze	0	0	0	1	0	2	0	0	2	1
Gloomy Thoughts	0	0	0	0	0	2	0	0	1	1

Data indicate number of subjects reporting an effect among a total of 27 subjects.

Table S3. Drug dose identification during session, after session and after the study

	Placebo		Low infusion (0.6 mg/min)		High Perfuison (1 mg/min)		Low bolus (15 mg) + low infusion (0.6 mg/min)		High bolus (25 mg) + high infusion (1 mg/min)	
	After session	After Study	After session	After study	After session	After study	After session	After study	After session	After study
correctly identified	96%	100%	67%	89%	85%	89%	78%	96%	81%	96%
missclassified as placebo			7%	0%	0%	0%	4%	0%	0%	0%
missclassified as low infusion (0.6 mg/min)	4%	0%			15%	11%	0%	0%	0%	0%
missclassified as high infusion (1 mg/min)	0%	0%	26%	11%			0%	0%	0%	0%
missclassified as low infusion (0.6 mg/min) + low bolus (15 mg)	0%	0%	0%	0%	0%	0%			19%	4%
missclassified as high infusion (1 mg/min) + high bolus (25 mg)	0%	0%	0%	0%	0%	0%	19%	4%		

After session = 1h after stop of the infusion; After study = at the end of study visit; n = 27.

Table S4. Additional pharmacokinetic parameters for DMT based on non–compartmental analyses [geometric mean (95% CI), range]

	Low infusion (0.6 mg/min)	High infusion (1.0 mg/min)	Low bolus (15 mg) + low infusion (0.6 mg/min)	High bolus (25 mg) + high infusion (1.0 mg/min)
AUC_{150} (ng*min/ml)	1369 (1170–1603)<br>552–3372	2297 (1961–2691)<br>984–5760	1899 (1650–2185)<br>836–3207	3213 (2883–3581)<br>1880–5244
AUC_{150} per mg ((ng*min/ml)/mg)	25 (22–30)<br>10–62	26 (22–30)<br>11–64	28 (24–32)<br>12–46	28 (25–31)<br>16–46
AUC $t_{1/2\alpha}$ (ng*min/ml)	116 (101–134)<br>68–259	200 (172–233)<br>78–450	117 (102–136)<br>58–344	198 (168–233)<br>91–618
AUC $t_{1/2\beta}$ (ng*min/ml)	33 (27–42)<br>8.6–75	58 (45–74)<br>14 –187	33 (26–41)<br>5.4–86	67 (53–84)<br>19–275
AUC $t_{1/2\beta}$ fraction (%)	22 (18–26)<br>9.7–43	21 (17–26)<br>5.0–59	21 (18–25)<br>6.6–39	25 (21–29)<br>8.0–56
Start time of $t_{1/2\alpha}$ (min)*	90	90	90	90
End time of $t_{1/2\alpha}$ (min)	106 (104–107)<br>100–120	107 (105–109)<br>100–120	106 (104–108)<br>100–120	107 (105–109)<br>100–120
Start time of $t_{1/2\beta}$ (min)	107 (105–109)<br>100–120	108 (106–109)<br>100–120	107 (105–109)<br>100–120	108 (106–110)<br>100–120
End time of $t_{1/2\beta}$ (min)$^{\Delta}$	143 (140–147)<br>120–150	148 (146–150)<br>130–150	142 (138–145)<br>120–150	148 (147–150)<br>130–150
DMT concentration at the end of $t_{1/2\alpha}$ (ng/ml)	2.3 (2.0–2.6)<br>1.2–4.1	3.8 (3.1–4.7)<br>1.4–11.3	2.3 (1.9–2.7)<br>0.95–4.7	4.2 (3.4–5.1)<br>1.5–12.0
DMT concentration at the start of $t_{1/2\beta}$ (ng/ml)	2.2 (1.8–2.6)<br>0.89–4.1	3.5 (2.8–4.3)<br>1.2–11.3	2.1 (1.8–2.6)<br>0.8–4.7	3.9 (3.2–4.8)<br>1.5–12.0

AUC, area under the plasma concentration-time curve; AUC_{150}, from time 0-150; AUC $t_{1/2\alpha}$, AUC from start to end time of $t_{1/2\alpha}$; AUC $t_{1/2\beta}$, AUC from start to end time of $t_{1/2\beta}$; AUC $t_{1/2\beta}$ fraction (%), fraction of AUC $t_{1/2\beta}$ to AUC $t_{1/2\alpha}$ + $t_{1/2\beta}$; n = 27

* equals to the timepoint of the infusion stop

$^{\Delta}$ equals to the timepoint of the last concentration measurement above the limit of quantification of 0.25 ng/ml DMT

Table S5. Tukey post hoc comparison of subjective and autonomic effects of DMT and placebo.

		Pla vs. LI	Pla vs. HI	Pla vs. LB + LI	Pla vs. HB + HI	LI vs. HI	LI vs. LB + LI	LI vs. HB + HI	HI vs. LB + LI	HI vs. HB + HI	LB + LI vs. HB + HI
Subjective Effects											
Any drug effect	ΔE_{max}	***	***	***	***	***	***	***	**	***	NS
	AUEC	***	***	***	***	***	NS	***	**	NS	***
	T_{max} (min)	***	***	NS	NS	NS	***	***	***	***	NS
Good drug effect	ΔE_{max}	***	***	***	***	**	***	***	NS	*	NS
	AUEC	***	***	***	***	**	NS	**	NS	NS	NS
Bad drug effect	ΔE_{max}	NS	NS	***	***	NS	***	***	**	***	NS
	AUEC	NS	NS	NS	***	NS	NS	**	NS	NS	NS
Fear	ΔE_{max}	NS	NS	**	**	NS	*	**	*	**	NS
	AUEC	NS	NS	NS	***	NS	NS	***	NS	***	**
Autonomic Effects											
Systolic blood pressure (mmHg)	E_{max}	NS	NS	***	***	NS	***	***	*	***	NS
Diastolic blood pressure (mmHg)	E_{max}	NS	*	***	***	NS	NS	***	NS	***	*
Heart rate (beats/min)	E_{max}	NS	NS	***	***	NS	***	***	**	***	NS
RPP (mmHg x bpm)	E_{max}	NS	NS	***	***	NS	***	***	***	***	NS
Acute adverse effects (LC)	ΔLC score	NS	NS	NS	**	NS	NS	NS	NS	NS	NS
Hormones											
BDNF (pg/ml)	Δ90 min	-	-	-	-	-	-	-	-	-	-
	Δ150 min	-	-	-	-	-	-	-	-	-	-
Oxytocin (pg/ml)	Δ60 min	NS	*	NS	NS	NS	NS	NS	NS	NS	NS

*p<0.05, **p<0.01, ***p<0.001; NS, not significant; AUEC, Area under the effect curve; BDNF, Brain-derived neurotrophic factor; Δ90min, difference from baseline concentration at 90 minutes; Δ120min, difference from baseline at 120 minutes; ΔEmax, maximal effect difference from baseline; HB, High bolus; HI, High infusion; LB, Low bolus; LI, Low infusion; LC, list of complaints; ΔLC score, difference of LC score from Baseline; RPP, Rate pressure product; Tmax, time to reach Emax; n = 27.

References

1. Riba, J., et al., *Metabolism and urinary disposition of N,N-dimethyltryptamine after oral and smoked administration: a comparative study.* Drug Test Anal, 2015. **7**(5): p. 401-6.
2. Holze, F., et al., *Pharmacokinetics and subjective effects of a novel oral LSD formulation in healthy subjects.* Br J Clin Pharmacol, 2019. **85**: p. 1474-83.
3. Dittrich, A., *The standardized psychometric assessment of altered states of consciousness (ASCs) in humans.* Pharmacopsychiatry, 1998. **31 (Suppl 2)**: p. 80-4.
4. Studerus, E., A. Gamma, and F.X. Vollenweider, *Psychometric evaluation of the altered states of consciousness rating scale (OAV).* PLoS One, 2010. **5**(8): p. e12412.
5. Liechti, M.E., P.C. Dolder, and Y. Schmid, *Alterations in conciousness and mystical-type experiences after acute LSD in humans.* Psychopharmacology, 2017. **234**: p. 1499-1510.
6. Carhart-Harris, R.L., et al., *The paradoxical psychological effects of lysergic acid diethylamide (LSD).* Psychol Med, 2016. **46**: p. 1379-90.
7. Schmid, Y., et al., *Acute effects of lysergic acid diethylamide in healthy subjects.* Biol Psychiatry, 2015. **78**(8): p. 544-53.
8. Dolder, P.C., et al., *LSD acutely impairs fear recognition and enhances emotional empathy and sociality.* Neuropsychopharmacology, 2016. **41**: p. 2638-2646.
9. Holze, F., et al., *Distinct acute effects of LSD, MDMA, and D-amphetamine in healthy subjects.* Neuropsychopharmacology, 2020. **45**(3): p. 462-471.
10. Bershad, A.K., et al., *Acute subjective and behavioral effects of microdoses of lysergic acid diethylamide in healthy human volunteers.* Biol Psychiatry, 2019. **86**(10): p. 792-800.
11. Preller, K.H., et al., *The fabric of meaning and subjective effects in LSD-induced states depend on serotonin 2A receptor activation* Curr Biol, 2017. **27**: p. 451-57.
12. Roseman, L., D.J. Nutt, and R.L. Carhart-Harris, *Quality of acute psychedelic experience predicts therapeutic efficacy of psilocybin for treatment-resistant depression.* Front Pharmacol, 2017. **8**: p. 974.
13. Griffiths, R.R., et al., *Psilocybin produces substantial and sustained decreases in depression and anxiety in patients with life-threatening cancer: a randomized double-blind trial.* J Psychopharmacol, 2016. **30**(12): p. 1181-1197.
14. Griffiths, R.R., et al., *Psilocybin can occasion mystical-type experiences having substantial and sustained personal meaning and spiritual significance.* Psychopharmacology (Berl), 2006. **187**(3): p. 268-83; discussion 284-292.
15. Barrett, F.S., M.W. Johnson, and R.R. Griffiths, *Validation of the revised Mystical Experience Questionnaire in experimental sessions with psilocybin.* J Psychopharmacol, 2015. **29**(11): p. 1182-90.
16. MacLean, K.A., M.W. Johnson, and R.R. Griffiths, *Mystical experiences occasioned by the hallucinogen psilocybin lead to increases in the personality domain of openness.* J Psychopharmacol, 2011. **25**(11): p. 1453-61.
17. Griffiths, R.R., et al., *Psilocybin occasioned mystical-type experiences: immediate and persisting dose-related effects.* Psychopharmacology, 2011. **218**(4): p. 649-65.
18. Griffiths, R., et al., *Mystical-type experiences occasioned by psilocybin mediate the attribution of personal meaning and spiritual significance 14 months later.* J Psychopharmacol, 2008. **22**(6): p. 621-32.
19. Garcia-Romeu, A., R.R. Griffiths, and M.W. Johnson, *Psilocybin-occasioned mystical experiences in the treatment of tobacco addiction.* Curr Drug Abuse Rev, 2014. **7**(3): p. 157-64.
20. Garcia-Romeu, A., et al., *Cessation and reduction in alcohol consumption and misuse after psychedelic use.* J Psychopharmacol, 2019. **33**(9): p. 1088-1101.
21. Griffiths, R.R., et al., *Psilocybin-occasioned mystical-type experience in combination with meditation and other spiritual practices produces enduring positive changes in*

psychological functioning and in trait measures of prosocial attitudes and behaviors. J Psychopharmacol, 2018. **32**: p. 49-69.
22. Ross, S., et al., *Rapid and sustained symptom reduction following psilocybin treatment for anxiety and depression in patients with life-threatening cancer: a randomized controlled trial.* J Psychopharmacol, 2016. **30**(12): p. 1165-1180.
